# COVID-19 Breakthrough Infections in Immune-Mediated Inflammatory Diseases: Data from the SUCCEED (Safety and Immunogenicity of COVID-19 Vaccines in Systemic Autoimmune-Mediated Inflammatory Diseases) Study

**DOI:** 10.3390/vaccines13020104

**Published:** 2025-01-22

**Authors:** Jeremiah Tan, Sasha Bernatsky, Jennifer L. F. Lee, Paul R. Fortin, Roya M. Dayam, Anne-Claude Gingras, Ines Colmegna, Dawn M. E. Bowdish, Claudie Berger, Dora Chan, Maggie J. Larché, Dawn P. Richards, Lourdes Gonzalez Arreola, Carol A. Hitchon, Nadine Lalonde, J. Antonio Aviña-Zubieta

**Affiliations:** 1 Arthritis Research Canada and Division of Rheumatology, University of British Columbia, Vancouver, BC V5Y 3P2, Canada; 2 The Research Institute of the McGill University Health Centre, Montreal, QC H3A 0E9, Canada; 3 Division of Rheumatology, Department of Medicine, McGill University, Montreal, QC H3A 0E9, Canada; 4 Centre de Recherche ARThrite—UL, Division of Rheumatology, Department of Medicine, CHU de Québec, Université Laval, Québec City, QC G1V 4G2, Canada; 5 Axe Maladies Infectieuses et Immunitaires, Centre de Recherche du CHU de Québec, Université Laval, Québec City, QC G1V 4G2, Canada; 6 Lunenfeld-Tanenbaum Research Institute at Mount Sinai Hospital, Sinai Health, Toronto, ON M5G 1X5, Canada; 7 McMaster Immunology Research Centre, McMaster University, Hamilton, ON L8S 4L8, Canada; 8 Division of Rheumatology, Department of Medicine, McMaster University, Hamilton, ON L8N 3Z5, Canada; 9 Canadian Arthritis Patient Alliance, Toronto, ON M6R 2J6, Canada; 10 Department of Internal Medicine, Max Rady College of Medicine, Rady Faculty of Health Sciences, University of Manitoba, Winnipeg, MB R3A 1R9, Canada

**Keywords:** COVID-19, vaccination, immune-mediated inflammatory diseases

## Abstract

**Background**: The Safety and Immunogenicity of COVID-19 Vaccines in Systemic Autoimmune-Mediated Inflammatory Diseases (SUCCEED) study was created to better understand COVID-19 vaccination in immune-mediated inflammatory disease (IMID). Knowing the frequency of COVID-19 breakthrough infections is important, particularly in IMID. Our objective was to assess these events in IMID. **Methods**: We prospectively studied IMID participants who had received ≥three COVID-19 vaccine doses. Individuals provided saliva samples monthly (September 2022 to August 2023). These were evaluated by polymerase chain reaction (PCR) for SARS-CoV-2. We also assessed antibodies against SARS-CoV-2 (anti-spike, SmT1, receptor binding domain, RBD, and nucleocapsid, NP) based on dried blood spots. Multivariable general estimating equation regression produced odd ratios (OR) for PCR SARS-CoV-2 positivity, related to demographics, immunosuppressives, and antibody levels. **Results**: Diagnoses included rheumatoid arthritis RA (*N* = 161, 44% of the total), systemic lupus, psoriatic arthritis, spondylarthritis, vasculitis, systemic sclerosis, and inflammatory bowel disease. Of the 366 participants, most were taking immunosuppressive medication. Of 1266 saliva samples, 56 (5.1%) were positive for SARS-CoV-2 on PCR. Higher anti-SmT1 antibodies were inversely associated with SARS-CoV-2 detection on PCR (adjusted OR 0.66, 95% confidence interval 0.45–0.97). Antibodies to SmT1, RBD, and NP were correlated and thus could not be included in a single model, but when anti-RBD was used in place of anti-SmT1, the results were similar. No other factor (including prior COVID-19 infection) was clearly associated with SARS-CoV-2 detection. **Conclusions**: This is the first study of SARS-CoV-2 in a large prospective cohort of triple (or more) vaccinated individuals with IMIDs. Anti-SmT1 antibodies appeared to be protective against later SARS-CoV-2 positivity, although recent past infection was not clearly related. This suggests the importance of maintaining robust vaccine-induced immunity through vaccination in IMID.

## 1. Introduction

### Background

COVID-19 vaccines have been shown to be effective in the general population [1]. However, COVID-19 breakthrough infections commonly occur, especially in people on immunosuppressive medications [2]. Several studies have demonstrated less durable antibody responses in people with immune-mediated inflammatory diseases (IMIDs) on immunosuppressives [3,4], compared to the general population. In part due to these issues, current recommendations suggest three COVID-19 vaccine doses represent the “primary series” in immunocompromised individuals (as opposed to in the general population, where two vaccine doses are considered the primary COVID-19 vaccination series [5].

COVID-19 breakthrough infections refer to cases where individuals, who have been fully vaccinated, become infected with the virus. Some infections are expected, as no vaccine is completely effective. However, knowing the frequency and impact of breakthrough infections is important, particularly in vulnerable subsets such as the immunocompromised. In Canada (as elsewhere), the first mRNA vaccine campaign in 2021 was highly effective at preventing severe outcomes from COVID-19. However, with the spread of more transmissible variants of concern, and waning immunity over time, breakthrough infections became more common, especially in the face of reduced immunity.

The Canadian COVID Immunization Task Force (CITF) was established to guide the Canadian government’s strategy on COVID-19 immunizations. The task force’s Vaccine Surveillance Reference Group played a critical role in ensuring that decision-makers had relevant data on vaccine safety and effectiveness. This enabled the Public Health Agency of Canada and the National Advisory Committee on Immunization to tailor recommendations for special populations, such as immunocompromised individuals and those with underlying health conditions like IMIDs. As part of their efforts, the CITF funded the Safety and Immunogenicity of COVID-19 Vaccines in Systemic Autoimmune-Mediated Inflammatory Diseases (SUCCEED) research program. This centres around COVID-19 immunity in people living with IMIDs, including systemic lupus erythematosus (SLE), rheumatic arthritis (RA), psoriatic arthritis (PsA), spondylarthritis (SpA), and other autoimmune diseases.

In the current paper, we present analyses of people living with IMID who had received three or more COVID-19 vaccine doses (their primary series). We evaluated potential demographic and clinical risk factors of “breakthrough” COVID-19 infection via reverse transcription quantitative polymerase chain reaction (PCR) testing on monthly saliva samples.

## 2. Methods and Materials

Our longitudinal, prospective, active surveillance study was conducted at four Canadian institutions in Quebec City, Montreal, Hamilton, and Vancouver. Participants were recruited between September 2022 and March 2023 consecutively, as they presented to these tertiary care rheumatology centres. They were initially invited to participate by their clinic rheumatology team, then (if they expressed interest), a research assistant on-site obtained their consent to participate in the study. We also contacted patients who had participated in prior IMID studies at these centres, who had previously agreed to be contacted for future research studies. Participants contributing to the serology assessment of the SUCCEED study provided dried blood spot (DBS) samples at regular intervals following each COVID-19 vaccine. These DBS samples were analyzed for anti-spike (SmT1), receptor binding domain (anti-RBD), and nucleocapsid antibodies.

To assess the effects of factors on “breakthrough” COVID-19 infection, we first (as noted in the introduction) defined the outcome based on the identification of SARs-CoV-2 using PCR testing on monthly saliva samples, as explained below. We then considered the above-described serology as covariates in our model, and also included demographic and clinical information, as noted in the following sections. To answer the question of whether these variables were associated with the presence of SARs-CoV-2 on PCR testing, we used general estimating equation (GEE) multivariate models to produce odds ratio (OR, with 95% confidence intervals, CI) estimates of the effects of each one of our variables on the outcome. For a given model covariate (i.e., serology, demographics, clinical factors), if an OR estimate was below the null value (1.0) with a 95% CI that also excluded the null value, we interpreted the evidence to be consistent with a negative association between the covariate and the outcome (SARs-CoV-2 positivity on saliva). That is, that covariate would be considered potentially as a factor associated with decreasing risk of “breakthrough” COVID-19 infection.

### 2.1. Saliva Samples

Once informed consent was provided, participants were given instructions and were mailed saliva collection kits and a questionnaire for each month asking about recent COVID-19 testing and symptoms in the prior 4 weeks. The saliva samples were obtained using the self-collection device DNA Genotek OMNIgene^®^ ORAL (OME-505, DNA Genotek Inc., Ottawa, ON, Canada) [6,7]. At one sitting, patients spit directly into this container for several minutes until the required 1.0 milliliter volume of saliva had been collected; they were asked during this time not to eat, drink, or smoke. The participant then sent the sample by mail to the principal investigator in Montreal.

Participants self-collected saliva samples in this manner, once a month for up to 6 months, sending the sample and completed questionnaires to the co-ordinating centre each month. If participants did not send their specimen/questionnaire on time, a reminder email or call was provided. All samples were sent to the McGill Genome Centre in Montreal, Canada, where samples were deactivated by a short incubation period at 50 °C and stored at −80 °C until RNA extraction.

The details of the detection of SARS-CoV-2 nucleic acid through multiplex PCR from the saliva samples are provided in Appendix A. In summary, viral RNA was extracted in batches (190 samples) from the saliva samples using RNAdvance Viral XP Reagent Kit (Beckman-Coulter, C59543) and each sample was tested twice with the Luna^®^ SARS-CoV-2 PCR Multiplex Assay Kit (NEB, E3019). Processing controls were also added to validate the test, including negative controls to monitor potential contamination through the whole sample processing and a positive control to evaluate validity of the real-time PCR assay. These tests qualitatively detect SARS-CoV-2 nucleic acid (specifically, two versions of N-gene fluorophores– N1 and N2) in the sample, indicating the presence of SARS-CoV-2. Our outcome of interest was saliva samples testing positive for SARS-CoV-2 (both N1 and N2 positive).

### 2.2. DBS Cards

Participants provided baseline information on past COVID-19 infection, COVID-19 vaccinations (including dates and type) and clinical history (type of IMID, medications). The SUCCEED protocol included collection of data and DBS at enrolment and at 2–4 weeks and 3, 6, and 12 months after COVID-19 vaccination. Participants were also contacted at 3, 6, and 12 months to update information on medications and confirm whether they had experienced additional COVID-19 infections or vaccinations. If participants had a new vaccination during follow-up, they reverted back to collecting biospecimens for serological sampling at 2–4 weeks and 3, 6, and 12 months post-vaccination, up to end of sampling. DBS collected by participants at home were mailed (in prepaid envelopes) back to each participating site and sent in batches to a lab for antibody testing. In brief, samples processed in the Gingras lab were tested with automated enzyme-linked immunosorbent assays for antibodies to the spike trimer (SmT1), the receptor binding domain (anti-RBD) and nucleocapsid using standardized assays [8].

The spike surface and core nucleocapsid proteins are the main immunogens of coronaviruses. The majority of available SARS-CoV-2 test kits and vaccines exploit the interaction between the SARS-CoV-2 spike glycoprotein and human anti-spike antibodies (with two sub-domains: S1 and the receptor binding domain—RBD). SmT1 is a reference material designed for the development of SARS-CoV-2 spike glycoprotein detection methods, as well as an antigen source for use in SARS-CoV-2 immunological assays. Anti-spike or anti-RBD antibodies are humoral immune markers generated post-vaccination or infection. Antibodies against the nucleocapsid protein have high sensitivity and specificity when used at least 14 days after infection and are not elicited by COVID-19 vaccines that target the spike protein or RBD protein.

### 2.3. Other Risk Factors and Covariables

Variables of potential importance in terms of their effects on COVID-19 infection included age (continuous), sex, race/ethnicity (white/non-white), autoimmune diagnosis (RA, SLE, other). The duration of IMID was categorized as: less than 5 years, 5 to less than 20 years, and ≥20 years. Self-reported recent past COVID-19 infection (binary) was defined as an infection (confirmed by home or lab testing) in the 6 months prior to the saliva sample (not counting the last 30 days before sample collection since we meant this variable to reflect past, not active infection). We defined the number of COVID-19 vaccines as 3–4 versus 5+ doses, and classified the time elapsed since their last COVID-19 vaccine as a categorical variable (<181 vs. ≥181 days, since the literature suggests waning of post-vaccine titres at this point). Vaccines received during this period were mRNA BNT162b2 (Comirnaty) and Spikevax. Prednisone use at the time of enrolment was dichotomized (yes/no). The use of immunosuppressive medication at enrolment was also dichotomized (yes/no), including biologics.

### 2.4. Statistical Analysis

We used counts, means, and medians to describe the characteristics of the participants at first saliva sample. Univariate and multivariable GEE models, with an autoregressive correlation structure, were created to study the association of testing positive for SARS-CoV-2, and potential factors of importance, including anti-SmT1, anti-RBD and anti-nucleocapsid antibodies. Multivariable GEE models were adjusted for sex, age, recent past SARS-CoV-2 infection, first line or biologic immunosuppressive medication use and number of days since the last COVID-19 vaccine.

All analyses were conducted using SAS Studio release 3.8 (2012–2018, SAS Institute Inc., Cary, NC, USA.)

The study was conducted according to the guidelines of the Declaration of Helsinki and approved by the respective research ethics committee at each participating site. In British Columbia, this study was approved by the University of British Columbia Clinical Research Ethics Board, under application H21-01391. In Ontario, this study was approved by the Hamilton Integrated Research Ethics Board, under project #13307. In the province of Quebec, both study sites were approved by the McGill University Health Centre Research Ethics Board, under the project #MP-37-2022-7763.

## 3. Results

### 3.1. Descriptive Statistics

We studied 366 participants with IMID who provided valid saliva samples between September 2022 and August 2023. Our participants initially collected 1918 saliva samples, but 652 saliva samples could not be included because they were found to be of insufficient quantity or quality and/or the DBS results were outside the required window. Thus, we were left with a total of 1266 valid saliva samples from 366 individuals to analyze.

Participants’ mean age at first sample was 56.7 (standard deviation 13.8) years, and most participants were female (79.8%) and White (85.5%). The IMID diagnoses were RA (*N* = 161, 44.0% of the total), SLE (*N* = 110, 30.1%), PSA (*N* = 58, 15.9%), SpA (*N* = 13, 3.6%), systemic sclerosis (*N* = 8, 2.2%), vasculitis (*N* = 10, 2.7%), and inflammatory bowel disease IBD (*N* = 6, 1.6%). The median duration since diagnosis was 12.1 years (interquartile range, IQR 5.7–23.1, Table 1). The majority of the participants were from Quebec (75%); 16% were from British Columbia and 9% from Ontario.

Those that reported a COVID-19 infection in the prior 6 months before baseline (excluding the 30 days before the first saliva sample) represented 12.8% of participants.

The most common baseline comorbidities included high blood pressure in 30.5% of individuals, asthma in 9.3%, diabetes in 8.9%, renal disease in 6.5%, and heart disease in 5.3%.

Of these 366 participants, 175 (47.8%) were on prednisone at baseline. Most (83.6%) of the participants were on baseline immunosuppression; some individuals were on combination therapy (e.g., hydroxychloroquine and/or a disease-modifying agent including biologics).

Among the 366 participants, 137 (37.4%) were on hydroxychloroquine at baseline, 142 (38.8%) on methotrexate, 12 (3.3%) on azathioprine, 11 (3.0%) on a Janus Kinase inhibitor, 20 (5.5%) on leflunomide, 3 on mycophenolate, and 1 on cyclosporin. In addition, there were 43 individuals on rituximab (11.8%), 36 on adalimumab (9.8%), 15 on secukinumab (4.1%), 13 on abatacept (3.6%), 11 on etanercept (3%), 10 on golimumab (2.7%), 9 on certolizumab (2.5%), 5 on ustekinumab (1.4%), 3 on infliximab, and 3 on tocilizumab.

At the first saliva sample, 90 (25%) had received three COVID-19 vaccine doses, The prior vaccination type for most participants was mixed monovalent (*n* = 119, 32.5%) followed by mixed bivalent (*n* = 108, 29.5%), Pfizer monovalent (*n* = 96, 26.2%) and Moderna monovalent (*n* = 43, 11.8%). The median for days since their last COVID-19 vaccination was 173 days with an interquartile range of 81–284. There were 193 participants (52.7%) for whom the last COVID-19 vaccination was less than 181 days and 173 (47.3%) more than 180 days ago.

The 366 participants provided 1266 saliva samples, and 65 samples (from 56 participants) were positive for SARS-CoV-2 PCR. Thus overall, 15.3% (56/366) participants tested positive at least once. Nine participants tested positive twice. Table 1 describes participants and their relevant demographic and clinical factors at the time of their first saliva sample, stratified by SARS-CoV-2 results (those with at least one positive test, and those who were never positive).

At entry into the study, the two groups did not clearly differ in terms of age, number of vaccine doses, past vaccine types, or previous SARS-CoV-2 infection. The characteristics of the participants stratified by saliva sample are described in Supplemental Table S1. Indicated in Supplemental Table S2, the median anti-SmT1 and anti-RBD levels before the first saliva test were 1721 (267–1721) BAU/mL and 2113 (626–6539) BAU/mL, respectively.

### 3.2. Results of Univariate and Multivariate GEE Models

The results of the univariable GEE models are presented in Supplemental Table S3. Except for anti-SmT1 antibodies, none of the model covariates were clearly associated with a positive SARS-CoV-2 saliva PCR test. Anti-SmT1 and anti-RBD antibodies were too correlated to be included in the same multivariable model. Since models with anti-SmT1 showed a better model fit compared to models with anti-RBD, anti-SmT1 was included in the final multivariable models (Table 2). Anti-SmT1 antibodies were negatively associated with positive PCR tests, such that each increase in 1000 BAU/mL was associated with a 34% decrease in the adjusted odds ratio of a positive PCR (OR 0.66, 95% confidence interval 0.45–0.97). There was no clear relationship between positive SARS-CoV-2 saliva PCR test and male sex (multivariate OR 0.93, 95% CI 0.46; 1.91), age (multivariate OR 1.02, 95% CI 0.93; 1.13), recent SARS-CoV-2 infection (multivariate OR 1.26, 95% CI 0.67; 2.38) immunosuppressives including biologics (multivariate OR 1.37 (95% CI 0.66; 2.87) or time since last COVID-19 vaccine (multivariate OR 1.42, 95% CI 0.81; 2.49).

## 4. Discussion

In this active surveillance study, we tested 1266 saliva samples from 366 individuals with IMID for SARS-CoV-2 positivity. Those with higher immune responses to vaccination had a lower OR for positive SARS-CoV-2 testing.

Early studies suggested the benefit of vaccination in terms of preventing severe COVID-19 in people with IMIDs [9,10]. Immunosuppressed individuals may be more susceptible to severe COVID-19, including hospitalization, ICU admission, and mortality [11].

Several studies have shown an impaired immunogenic response to COVID-19 vaccines in IMID patients [12,13], which has led authorities to recommend that the primary series in immunosuppressed people with IMID should be three doses, as opposed to the “two-dose” primary series for mRNA COVID-19 vaccination in the general population [14,15,16].

Nearly all previous studies assessing COVID-19 infection rates in IMID are retrospective cohort studies. These may have missed individuals who had mild symptoms or indeed were asymptomatic. In contrast, we used active prospective surveillance to capture the prevalence of SARS-CoV-2 positivity in IMID.

Five percent of saliva samples in our cohort were positive for SARS-CoV-2. Between December 2020 and November 2021, a large-scale English retrospective general population cohort study assessed all confirmed COVID-19 test results (PCR and rapid antigen swab tests in symptomatic and asymptomatic individuals) and found a SARS-CoV-2-positivity rate of 3.8% after two COVID-19 vaccine doses [17]. A large American electronic health record analysis of over 150,000 immunocompromised individuals from January 2021 to March 2022 found a prevalence of 2.9% for breakthrough infection (based on administrative diagnostic codes) after two mRNA vaccine doses [18]. A nested case-control analyses of a population-based IMID Ontario (Canada) cohort (from March to November 2021) used nasal PCR test results to evaluate break-through infections. Authors estimated COVID-19 infection prevalence at 5.4–6.5% in IMID individuals who had received two mRNA COVID-19 vaccine doses [19].

Our multivariate analysis did not show any clear associations for SARS-CoV-2 positivity and sex, age, immunosuppression, prior recent COVID-19 infection, or time elapsed since last vaccine. Other studies have associated positive tests in IMID with various factors, but these were often in cohorts that had not received three or more vaccines. In one retrospective cohort study, conducted in 2021 at a large American multi-centre healthcare system in persons with rheumatic diseases who had received two doses of mRNA vaccine, breakthrough COVID-19 infections occurred more often in individuals using mycophenolate, methotrexate, rituximab, and tumor necrosis factor inhibitors [20].

Regarding immune responses after COVID-19 vaccination and protection against infection, this area remains uncertain. After vaccination, anti-RBD antibodies and anti-SmT1 antibodies may play different roles in terms of protecting against future COVID-19 infection. The receptor-binding domain of the spike protein of the SARS-CoV-2 virus is the part that binds to the ACE2 receptor on host cells, allowing the virus to enter, causing infection. Anti-RBD antibodies, by blocking this binding between the spike protein and ACE2 receptors, may prevent viral entry and reduce the likelihood of infection. High levels of anti-RBD antibodies may thus help lower the risk of infection and hospitalization. In contrast to anti-RBD antibodies, the role of antibodies to SmT1 (another component of the spike protein) is less clear.

There are distinct differences between our study and the aforementioned previous studies: first, our study is a prospective active surveillance study, whereas other studies were retrospective, usually recording testing done for symptomatic patients. Secondly, all previous estimates were based on only two vaccine doses, whereas we evaluated individuals with at least three doses (which is currently accepted as the primary series in immunosuppressed IMID patients). To our knowledge, ours is the first study to assess breakthrough COVID-19 infection in a large prospective cohort of triple+-vaccinated individuals with IMIDs.

While this study’s prospective, active surveillance design was a major strength, it did have some potential limitations. First, data were gathered across a selection of diseases and drug exposures. While this diversity may be beneficial to generalizability, it may obscure trends and relationships that would otherwise exist for specific patient groups. Specifically, with the relatively low number of SARS-CoV-2 positive results, it would be difficult to demonstrate subtle differences in risk related to specific medication exposures, or other potential risk factors.

Our study used saliva collection to assess for the presence of SARS-CoV-2, as it is more comfortable, less invasive, and in some ways easier to collect than nasal/nasopharyngeal swabs. However, saliva sampling potentially has lower sensitivity for SARS-CoV-2 detection than nasal/nasopharyngeal swabs, especially early on, or in the setting of low viral load. Saliva collection for SARS-CoV-2 detection may also pose problems in terms of more variability in sample quality, and indeed many of the samples were not of adequate quantity or quality for testing. However, though, nasal and nasopharyngeal swabs are a valuable method for SARS-CoV-2 detection, saliva collection was more appropriate for a surveillance study like ours, when we were aiming to recruit a relatively large number of asymptomatic individuals, and did not want to risk participants refusing on the basis of the requirement for the more invasive, uncomfortable nasal/nasopharyngeal swabs.

## 5. Conclusions

We analyzed 1266 saliva samples from 366 IMID participants who were vaccinated three+ times and found that 15.3% (*N* = 56) of participants tested positive for SARS-CoV-2 on PCR between September 2022 and August 2023. We were unable to detect significant differences in positivity based on sex, immunosuppressives medications, vaccine type, or time elapsed since last vaccination. The one factor that seemed to be protective against SARS-CoV-2 positivity on PCR was anti-SmT1 antibodies. Specifically, recent past infection did not seem to be protective against later SARS-CoV-2 positivity. These findings suggest the importance of maintaining high seroconversion with regular vaccination in immunosuppressed IMID individuals living with IMID.

## Figures and Tables

**Table 1:** Characteristic of participants at first saliva sample, overall and stratified by saliva result for SARS-CoV-2 at any time point.

Characteristic		All (*n* = 366)	Result of the Saliva Samples	
			*N* (%) Positive, Any Sample (*n* = 56)	*N* (%) Negative, All Samples (*n* = 310)
**COVID-19 infection: 6 months to 30 days before saliva test, *N* (%)**		47 (12.8)	8 (14.3)	39 (12.6)
**Age (years), mean (SD)**		56.7 (13.8)	59.6 (12.5)	56.2 (14.0)
**Female, *N* (%)**		292 (79.8)	45 (80.4)	247 (79.7)
**Caucasian, *N* (%)**		313 (85.5)	52 (92.9)	261 (84.2)
**Autoimmune Diagnosis, *N* (%)**	Rheumatoid arthritis	161 (44.0)	26 (46.4)	135 (43.6)
	Systemic lupus	110 (30.0)	13 (23.2)	97 (31.3)
	Other *	95 (26.0)	17 (30.4)	78 (25.2)
**Autoimmune disease duration, *N* (%)**	<5 years	71/363 (19.6)	14 (25.0)	57/307 (18.6)
	5 to <20	190/363(52.3)	31 (55.43)	159/307(51.8)
	20+ years	102/363(28.1)	11 (19.6)	91/307 (29.6)
**Baseline prednisone, *N* (%)**		175 (47.8)	28 (50.0)	147 (47.4)
**Immunosuppressives including biologics, *N* (%)**		252 (68.9)	44 (78.6)	208 (67.1)
**Calendar month of saliva test**	September to November 2022	111 (30.3)	20 (35.7)	91 (29.4)
	December 2022 to Febuary 2023	87 (23.8)	15 (26.8)	72 (23.2)
	March to May 2023	152 (41.5)	19 (33.9)	133 (42.9)
	June to August 2023	16 (4.4)	2 (3.6)	14 (4.5)
**Vaccine doses, *N* (%)**	3–4 doses	222 (60.7)	30 (53.6)	192 (61.9)
	5 doses	144 (39.3)	26 (46.4)	118 (38.1)
**Prior vaccine type *N* (%)**	Pfizer monovalent	96 (26.2)	14 (25.0)	82 (26.5)
	Moderna monovalent	43 (11.8)	4 (7.1)	39 (12.6)
	Mixed monovalent	119 (32.5)	18 (32.1)	101 (32.6)
	Mixed bivalent	108 (29.5)	20 (35.7)	88 (28.4)
**Days since last COVID-19 vaccination**	median (IQR **)	173 (81–284)	122 (72–240)	179 (86–290)
	<181 days, *n* (%)	193 (52.7)	19 (33.9)	154 (49.7)
	181 days+, *n* (%)	173 (47.3)	37 (66.1)	156 (50.3)

* Others included psoriatic arthritis (*n* = 58, 15.9), spondylarthritis (*n* = 13, 3.6), systemic sclerosis (*n* = 8, 2.2), vasculitis (*n* = 10, 2.7), and inflammatory bowel disease (*n* = 6, 1.6) ** IQR = interquartile range.

**Table 2:** Odds ratios, OR (95% confidence intervals, CI) for SARS-CoV2 detected by PCR on saliva univariable and multivariable GEE models *.

Covariates	Univariable OR (95% CI)	Multivariable OR (95% CI)
SmT1 (per 1000 BAU/mL)	0.63 (0.44; 0.92)	0.66 (0.45; 0.97)
Male sex	1.01 (0.51; 2.02)	0.93 (0.46; 1.91)
Age (per 5 years)	1.05 (0.95; 1.16)	1.02 (0.93; 1.13)
SARS-CoV2 infection: 6 months to 30 days before saliva test (Yes/no)	1.27 (0.67; 2.40)	1.26 (0.67; 2.38)
Immunosuppressives including biologics (Yes/no)	1.59 (0.83; 3.04)	1.37 (0.66; 2.87)
Number of days since last COVID-19 vaccine (<181 versus 181+ days)	1.36 (0.79; 2.34)	1.42 (0.81; 2.49)

* Auto-regressive correlation matrix.

## Data Availability

The data are contained within the article. The original contributions presented in the study are included in the article; further inquiries can be directed to the corresponding author.

## Supplementary Materials

The following supporting information can be downloaded at: https://www.mdpi.com/article/10.3390/vaccines13020104/s1, Table S1: Characteristics of the participants at each saliva sample; Table S2: Most recent DBS results stratified by saliva sample; Table S3: Odds ratios, OR (95% confidence intervals CI) for positive SARS-CoV-2 PCR saliva test (vs. negative) in univariable generalized estimation equation models.

## Appendix A

### PCR Method

**Collected and Storage**: Samples were collected using the self collection device DNA Genotek OMNIgene^®^ ORAL (OME-505) device (DNA Genotek Inc., Ottawa, ON, Canada) then sent by mail to the principal investigator in Montreal. Samples were sent to the McGill Genome Centre (MGC) less than 21 days post collection. Upon arrival to the MGC, samples are inactivated in a dry bath at 50 °C for 2 h then placed at −80 °C until extraction.

**Extraction**: On the day of extraction, samples are thawed at room temperature then plated in a 96-well format. A sample of 200 μL of saliva is transferred by the Janus Liquid Handler into a deep well plate containing 150 μL of Beckman Lysis Buffer from the RNAdvance Viral XP Extraction kit (cat# C59543). Extraction is automated on the Biomek I7 with HEPA enclosure.

**RT-qPCR**: RT-qPCR is carried out with the Luna^®^ SARS-CoV-2 RT-qPCR Multiplex Assay Kit from NEB (cat# E3019L) at half volume in 384 well plate. qPCR mastermix is dispensed into the plate using the Formulatrix Mantis liquid dispenser. Samples are added either with the BiomekI7 or the Formulatrix F.A.S.T liquid handler. The kit contains 3 probes; HEX (533) for the SARS-CoV2 N1 gene, FAM (465) for the SARS-CoV2 N2 gene and Cy5 (618) for the human RNAseP gene which serves as an internal control for sample quality. Amplification and readings are carried out on the LightCycler^®^480 Instrument II from Roche for a total of 45 cycles.

**Analysis**: Each run is analyzed using the derivative method first on each of the 3 probe filters. If the run is too noisy, i.e., with a large background band at the baseline or multiple “Step” increase in fluorescence that are confounded with actual amplification curves, then a secondary analysis by point threshold is applied. This secondary analysis requires more time but enables most runs to be resolved. A report of positive for samples having either N1 signal or N2 signal or both with Ct values lower than 40.
