# Supplementary material for: COVID-19 Breakthrough Infections in Immune-Mediated Inflammatory Diseases: Data from the SUCCEED (Safety and Immunogenicity of COVID-19 Vaccines in Systemic Autoimmune-Mediated Inflammatory Diseases) Study
Source: Vaccines (Basel). 2025 Jan 22;13(2):104. doi: 10.3390/vaccines13020104 (PMC11860671; doi:10.3390/vaccines13020104)
Supplement: Supplementary file 1 [file vaccines-13-00104-s001.zip › vaccines-3379939-supplementary.pdf]

Supplemental Table S1: Characteristics of the participants at each saliva sample.

Characteristics		Saliva sample					
		1st (n=366)	2nd (n=313)	3rd (n=244)	4th (n=173)	5th (n=121)	6th (n=49)
COVID-19 infection: 6 months to 30 days before saliva test, n (%)		47 (12.8%)	54 (17.3%)	56 (23.0%)	42 (24.3%)	37 (30.6%)	9 (18.4%)
Female, n (%)		292 (79.8%)	251 (80.2%)	197 (80.7%)	138 (79.8%)	96 (79.3%)	40 (81.6%)
Age (years), mean (SD)		56.7 (13.8)	57.0 (13.8)	57.8 (13.5)	58.3 (13.5)	59.1 (13.0)	60.1 (12.2)
Caucasian, n (%)		313 (85.5%)	272 (86.9%)	216 (88.5%)	155 (89.6%)	107 (88.4%)	45 (91.8%)
1st line or biologic medication at baseline, n (%)		252 (68.9%)	217 (69.3%)	177 (72.5%)	123 (71.1%)	84 (69.4%)	35 (71.4%)
Prednisone use, n (%)		175 (47.8%)	149 (47.6%)	115 (47.1%)	86 (49.7%)	64 (52.9%)	27 (55.1%)
Number of vaccine doses, n (%)	3-4	222 (60.7%)	176 (56.2%)	133 (54.5%)	87 (50.3%)	54 (44.6%)	14 (28.6%)
	5-6	144 (39.3%)	137 (43.8%)	111 (45.5%)	86 (49.7%)	67 (55.4%)	35 (71.4%)
Number of days since last COVID-19 vaccine, n (%)	< 181	193 (52.7%)	149 (47.6%)	112 (45.9%)	69 (39.9%)	48 (39.7%)	17 (34.7%)
	≥ 181	173 (47.3%)	164 (52.4%)	132 (54.1%)	104 (60.1%)	73 (60.3%)	32 (65.3%)

Supplement Table S2: Most recent DBS results stratified by saliva sample.

	Saliva sample					
	**1st**	**2nd**	**3rd**	**4th**	**5th**	**6th**
**Sample size for NP or SmT1**	359	309	243	172	121	49
**Time between DBS and saliva (days), median (IQR)**	28 (0-75)	46 (29-92)	62 (40-92)	77 (33-109)	81 (42-122)	73 (39-129)
**NP ≥ 34.6 BAU/ml, n (%)**	86 (24.0%)	77 (25.0%)	58 (23.9%)	45 (26.2%)	29 (24.0%)	11 (22.5%)
**NP (BAU/ml), median (IQR)**	9.4 (4.6-31.1)	10.2 (4.6-32.9)	10.5 (4.7-32.6)	10.5 (5.1-36.5)	10.5 (4.9-33.0)	11.6 (4.9-33.0)
**SmT1 ≥ 11.3 BAU/ml, n (%)**	350 (97.5%)	303 (98.1%)	241 (99.2%)	171 (99.4%)	120 (99.2%)	49 (100.0%)
**SmT1 (BAU/ml), median (IQR)**	1721 (267-1721)	1721 (797-1721)	1721 (970-1721)	1721 (1431-1721)	1721 (1721-1721)	1721 (1721-1721)
**Sample size for RBD**	366	313	244	173	121	49
**Time between DBS and saliva (days), median (IQR)**	29 (0-76)	47 (29-92)	62 (40-93)	78 (34-111)	81 (42-122)	73 (39-129)
**RBD ≥ 31 BAU/ml, n (%)**	345 (94.3%)	297 (94.9%)	236 (96.7%)	168 (97.1%)	116 (95.9%)	49 (100.0%)
**RBD (BAU/ml), median (IQR)**	2113 (626-6539)	2804 (690-6539)	2965 (818-6539)	3842 (1435-6539)	4316 (1561-6539)	4399 (1686-6539)
**RBD≥31 or SmT1≥11.3, n (%)**	356 (97.3%)	307 (98.1%)	242 (99.2%)	172 (99.4%)	120 (99.2%)	49 (100.0%)

Supplemental Table S3: Odds Ratios, OR (95% confidence intervals CI) for positive SARS-CoV-2 PCR saliva test (vs. negative) in univariable generalize estimation equation models.

		OR (95% CI)
**COVID-19 infection: 6 months to 30 days before saliva test**	Yes vs. no	1.27 (0.67; 2.40)
**Age** (per 1 year)		1.05 (0.95; 1.16)
**Sex**	Female vs male	1.01 (0.51; 2.02)
**Caucasian**	yes vs. no	0.56 (0.20; 1.61)
**Autoimmune Diagnosis**	SLE vs. RA	0.85 (0.43; 1.64)
	Other vs. RA	1.60 (0.86; 2.98)
**IMID Diagnosis Duration**	5 to < 20 vs. < 5 yrs	1.07 (0.57; 1.99)
	20 + vs. < 5 yrs	0.49 (0.23; 1.06)
**Immunosuppressive**	Yes vs. no	1.02 (0.45; 2.30)
**Prednisone use**	Yes vs. no	0.90 (0.53; 1.52)
**First line or biologic medication**	Yes vs. no	1.59 (0.83; 3.04)
**Date of saliva test**	Sept-Nov 2022 vs. June-August 2023	0.78 (0.31; 1.95)
	Dec 2022 Feb 2023 vs. June-August 2023	0.94 (0.48; 1.86)
	March-May 2023 versus June-August 2023	0.68 (0.36; 1.27)
**Prior vaccine doses**	3-4 vs. 5 doses	0.98 (0.58; 1.66)
**Prior vaccine type**	BNT162b monovalent vs. mixed bivalent	0.84 (0.42; 1.69)
	Moderna monovalent vs. any bivalent	0.62 (0.20; 1.86)
	Mixed monovalent vs. Mixed bivalent	0.88 (0.47; 1.65)
**Days since last COVID-19 vaccination**	< 181 vs. ≥ 181 days	1.36 (0.79; 2.34)
**Anti-spike** - dichotomous	Positive vs. negative*	0.45 (0.10; 1.94)
**Anti-spike** - continuous	per 1000 BAU/ml	0.63 (0.44; 0.92)
**Anti-RBD** - dichotomous	Positive vs. negative*	0.52 (0.19; 1.43)
**Anti-RBD** - continuous	per 1000 BAU/ml	0.90 (0.81; 1.01)
**Anti-NP** - dichotomous	Positive vs. negative*	1.23 (0.69; 2.20)
**Anti-NP** - continuous	per 100 BAU/ml	0.91 (0.80; 1.05)

*Anti-spike positive is defined as Anti-spike ≥ 11.3 BAU/ml. Anti-RBD positive is defined as Anti-RBD ≥ 31 BAU/ml. Anti-NP positive is defined as Anti-RBD ≥ ≥ 34.6 BAU/ml.
